# State Care of Idiots

**Published:** 1897-04

**Authors:** F. R. Martin

**Affiliations:** Kyle, Texas


For the Texas Medical Journal.

### STATE CARE OF IDIOTS.

BY F. R. MARTIN, M. D., KYLE, TEXAS.

[Read at Austin District Medical Society, December 21, 1896.]

THERE is a class of unfortunates in our State for which Texas has not, up to this time, made any provision. I have reference to idiots. This class of persons is quite numerous, probably averaging one to every fifteen hundred or two thousand of our population. They range from persons whose minds are but slightly impaired, to persons in whom mental perception or faculties are entirely absent. For the first I would not favor the State's providing any asylum facilities. Many of them are entirely inoffensive and agreeable, and are efficient in ordinary employments. But for idiots of the average or of the lowest degree of mental power, all the impulses of humanity demand the State's caring for them as it does the insane. And in support of this view I offer the following reasons:

First. Idiots, in the usual acceptation of the term, are the most irresponsible class of persons. They cannot be trusted in any relation of life, and for this reason, when one happens to be in a family of ordinary or, perhaps, stringent financial circumstances, he is a burden most grievously to be borne. His irresponsibility demands a constant care that cannot be given, only at the sacrifice of all the productive energy of some intelligent member. He cannot be left alone, for the injury he might bring to property by fire, etc., or the injury he might cause his own body by accident.

Moreover, it is a characteristic of idiots to be vulgar in their person, and for this reason families that have them are often humiliated and shamed beyond any reasonable patient endurance.

Furthermore, it is another characteristic of idiots, where they are not guarded as above mentioned, to rove over the community, thus causing the most serious embarrassment and apprehension to the families of the vicinity. They fear bodily harm being done to children and domestic animals, and they fear destruction to property by breakage or fire. To the timid an idiot at large is an object of dread and alarm, the unfortunate condition of his mind being invariably, by such persons, attributed to insanity.

Second. Idiots, as a class, demand environments entirely different from those demanded by rational persons. And I think it can be asserted, without fear of contradiction, that no family, or but few, at least, can produce those environments. Idiots, as a rule, are naturally of a cheerful turn of mind, and they require social attention and conversation suited to their range of mental vision. No family that has an idiot in it can, on account of the stern duties of life, adjust itself, only, perhaps, in a small degree, to this demand of the idiotic member. And again, some idiots, perhaps all, even of a very low degree of intelligence, are capable of some mental culture. But, as is the case with the deaf and dumb and the blind, it cannot be accomplished by the ordinary means of instruction. No family can, in the very nature of the case, undertake the task. I argue that if the State had justly undertaken to provide for the comfort, entertainment and culture of the deaf and dumb and the blind, it owes the same service to that more unfortunate class, the idiotic. The analogy ceases here, for the State should continue this service for an indefinite time or for life, as per my reason *first*, and others to follow.

Third. It is not infrequently the case that an idiot's parents are dead, and his care falls upon some relative or friend. This is a burden that all the dictates of an enlightened conscience forbids. No brother, sister, relative, or friend, unless he so elects, should be expected to carry it. Where no one has been found to care for the idiot, he has been put upon the county as a pauper, where there is little or no sympathy, no watchful or tender care; no restraint to prevent his inflicting injury upon himself or others. The counties are not now prepared to care for idiots, and, perhaps, never will be.

Fourth. Idiots, in a few cases, have been known to exhibit strong symptoms allied to insanity. In the radius of my acquaintance there is an instance that, in my opinion, falls within this class. This individual belongs to that classification of idiocy known as congenital, in which there is defective mental power, with other associated congenital conditions, as contra-distinguish from that form of idiocy in which there is an entire absence of mental power. The treatment and general management of this individual has been as judicious a combination of medical, physical and intellectual agencies as the circumstances of his family would permit. Up to three years ago, his condition was as good, probably, as that of the ordinary idiot, carrying with it, of course, all the inevitable attendant evils already referred to in this paper. Since three years ago, at which time he had measles, there has been a remarkable transformation. While his mental state has remained about the same as ever, his disposition seems to be altogether perverted. Before, he was obedient, docile and easily managed; now he is obstinate and unmanageable, persistent in his inclinations to do every thing in opposition to the wishes of those having his immediate care. He exhibits at all times striking evidences of monomania, for instance kleptomania, stealing every article that he can get hold of—pencils, papers, books, clothing, etc., showing while doing so all of those cunning and secretive characteristics of such a condition. His mania is likely to assume any form, more likely that of pyromania, which would make it extremely hazardous for him to run at large. He exhibits, also, violent outbreaks of anger, and he has on several occasions inflicted serious bodily injuries on children. It is hard to reach any other conclusion, contrasting this individual's present condition with his former, than that he is, in some sense, insane.

The parents of this idiot are dead, and he lives with a brother. Intimate acquaintance with the family enables me to say that I give it as my deliberate judgment, that if half of the insane now in the State's asylums were back at home, would not give the trouble that is being caused by this individual to his brother's family.

Fifth. I give it as my fifth and last reason, and there are probably others that could be given, why the State should provide asylum facilities for idiots, that other States have done so. While I have no official information on the subject, I think an investigation of the matter will show that many of the leading governments of the world, and that a number of the States of this Union, have made such provisions. Surely, Texas can no longer afford to be behind the foremost State in its work of humanity.

In conclusion, allow me to remark that I do not believe that if the suggestions herein made were enacted into a law it would impose a serious burden on the State. All idiots capable of any kind of useful employment, those who have parents or relatives able to care for them, and those harmless and decent in their deportment, would very probably be kept at home.

[Dr. Martin should cause this patient to be examined in the usual way, and if found to be insane, as he suspects, have him sent to the insane asylum.—Ed.]

